# Effect of mixed partial occupation of metal sites on the phase stability of γ-Cr_23**−***x*_Fe_*x*_C_6_ (*x* = 0–3) carbides

**DOI:** 10.1038/s41598-018-25642-y

**Published:** 2018-05-08

**Authors:** Maaouia Souissi, Marcel H. F. Sluiter, Tetsuya Matsunaga, Masaaki Tabuchi, Michael J. Mills, Ryoji Sahara

**Affiliations:** 1 Research Center for Structural Materials, National Institute for Materials Science, 1-2-1 Sengen, Tsukuba, Ibaraki, 305-0047 Japan; 2 Department of Materials Science and Engineering, Delft University of Technology, Mekelweg 2, 2628 CD Delft, The Netherlands; 3 Department of Materials Science and Engineering, The Ohio State University, 478 Watts Hall, 2041 College Rd, Columbus, OH 43210 USA

## Abstract

The effect of mixed partial occupation of metal sites on the phase stability of the γ-Cr_23−*x*_Fe_*x*_C_6_ (*x* = 0–3) carbides is explored as function of composition and temperature. Ab initio calculations combined with statistical thermodynamics approaches reveal that the site occupation of the carbides may be incorrectly predicted when only the commonly used approach of full sublattice occupation is considered. We found that the γ-M_23_C_6_ structure can be understood as a familiar sodium chloride structure with positively charged rhombic dodecahedron (M^(4a)^ M_12_^(48h)^) and negatively charged cubo-octahedron (M_8_^(32f)^ C_6_^(24e)^) super-ion clusters, together with interstitial metal atoms at the 8c sites. The stability of the partially occupied phase can be easily rationalized on the basis of a super-ion analysis of the carbide phase. This new understanding of γ-M_23_C_6_ carbides may facilitate further development of high-chromium heat-resistant steels.

## Introduction

The phase stability of carbide precipitates plays a crucial role in improving the creep lifetime of steels^1,2^. For instance, a high density of γ-M_23_C_6_ carbides (in which M denotes a metal element), increases the creep rupture strength, as grain boundary sliding and surface cracking are reduced. Although the chemical compositions of some of these carbides have been determined, experimental studies on their mechanism of formation, atomic site occupation, and chemical interactions between species in multicomponent phases have seldom been reported^3,4^.

The partial/full occupation of a sublattice site refers to the fraction of the site that is occupied by a particular atomic species, averaged over many unit cells. To date, the vast majority of ab initio total energy studies have considered only configurations in which sublattice sites were fully occupied by a single atomic species^5–19^, in particular with regard to Frank-Kasper structures^20^. When mixed occupancy of sublattice sites was considered it was only to the extent as necessitated by the cluster expansion method, say when nearest neighbor interactions between sites of the same type needed to be evaluated, such as is the case in the σ structure^21–24^. Mixed occupancy was considered in the specific context of the coherent potential approximation^25^, and for several configurations in the Fe-Cr σ structure^26^ but an in-depth analysis including associated effects such as due to lattice vibrations remains lacking. Here, we address this issue by considering many partially occupied configurations of a non-trivial technically important carbide phase, Cr_23_C_6_, while including vibrational effects.

**Figure 1:**
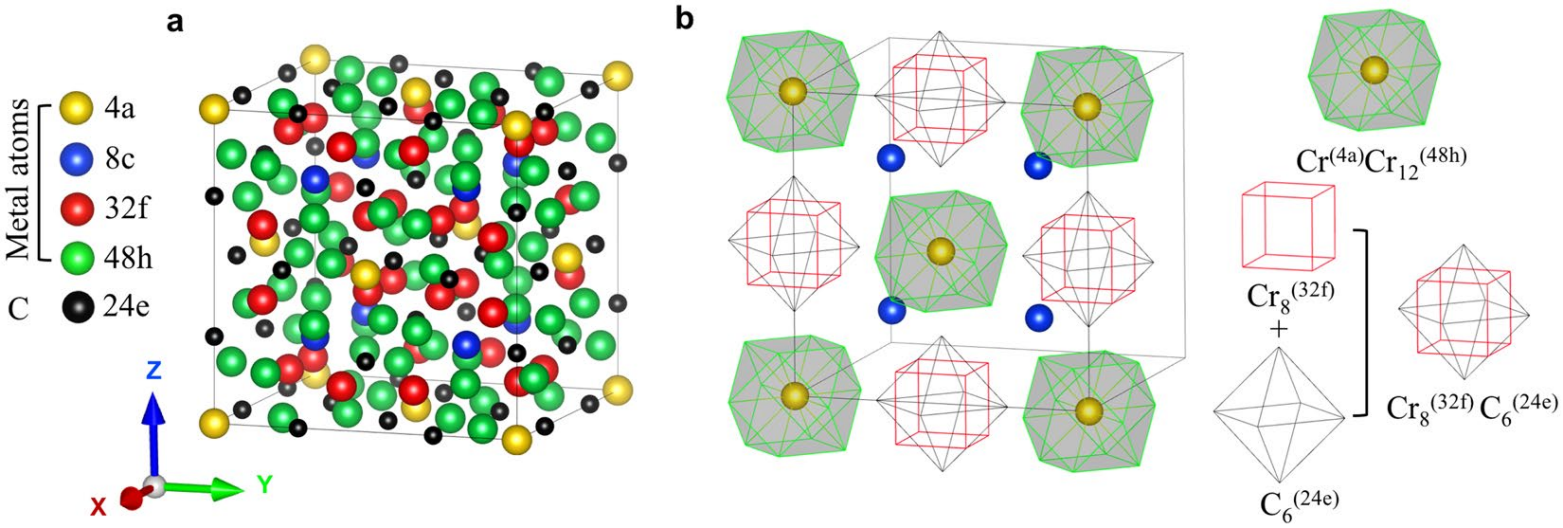
(**a**) The γ-Cr_23_C_6_ supercell consists of one conventional unit cells with four inequivalent metal sites, namely, the 4a, 8c, 32f, and 48h sites, according to Wyckoff notation. The C atoms are located at the 24e sites. (**b**) The carbide NaCl super-ion morphology and interstitial (8c) atomic arrangement in γ-Cr_23_C_6_. The supercell is constructed from four Cr^(4a)^Cr_12_^(48h)^ clusters and four Cr_8_^(32f)^C_6_^(24e)^ clusters, together with eight Cr atoms in the 8c sites.

The γ-M_23_C_6_ crystal has a face-centered cubic (fcc) structure with the $Fm\bar{3}m$ space group. The supercell contains four (M_23_C_6_) unit cells and consists of 92 metal atoms occupying four inequivalent metallic sites, namely, the 4a, 8c, 32f, and 48h sites, and 24 non-metallic atoms occupying the 24e sites, as represented using Wyckoff notation, according to the results of X-ray powder diffraction^27^. Figure 1a shows the crystal structure of γ-Cr_23_C_6_, and Fig. 1b shows a simplified view of the super-ion arrangement originally already recognized by Westgren^27^. In this structure, each 4a site is surrounded by 12 metal atoms in 48h sites, forming a cubo-octahedron with the composition Cr^(4a)^Cr_12_^(48h)^. On the other hand, the neighboring 32f sites form a cube, and protruding from the faces of the cube are the 24e sites that together form a concentric octahedron. The closely positioned 32f and 24e sites together form a rhombic dodecahedron with the composition Cr_8_^(32f)^C_6_^(24e)^ and eight Cr^(8c)^ atoms lie at the interstitial positions. In this work, we treat only the mixed, i.e. partial, occupation of the metallic sites, and not the C occupied sites. We suppose that C atoms are fully occupying the 24e sites. Partial occupancy of the carbon sublattice can be important in some carbides^28,29^, but in this carbide we do not expect it to play a role of significance because the carbon vacancy formation enthalpy is very large (~1.05 eV/C vacancy)^30^.

Experimentally, according to single-crystal X-ray diffraction data, there is evidence of the selective metallic site occupation of Fe in γ-Cr_23−*x*_Fe_*x*_C_6_ in the range of 0 ≤ *x* ≤ 7.36^31,32^. At a low Fe fraction, the Fe atoms occupy the 4a and 8c sites, and they simultaneously begin to occupy the 48h and 32f sites at higher Fe fractions. The occupancy sequence was found for 4a and 8c sites from atomistic simulations using pair potentials obtained through the lattice inversion technique^33^. However, simple pair potentials generally favor topologically close packed structures and generally cannot yield complex structures such as the present carbide as a true ground state. Therefore, in an MD simulation at finite temperatures, these pair potentials quickly lead to an unrealistic complete collapse of the crystal structure^33^. Moreover, the pair potentials do not account for changes in magnetic order, cannot reproduce elastic anisotropy, and fail to reproduce non-isotropic structural relaxations. Clearly, ab initio approaches which properly account for many-body interactions are much preferred for studies of the occupancy sequence in M_23_C_6_.

A few ab initio studies considered partial site occupation in M_23_C_6_. Medvedeva *et al*.^34^ considered Cr_22_Fe_1_C_6_, where Fe either fully occupies the 4a site, or partially occupies an 8c, 32f, or 48h site. It was found that Fe prefers to occupy the 4a site and that Cr_22_Fe_1_^(4a)^C_6_ is more stable than a mixture of Cr_23_C_6_ and Fe_23_C_6_^35,36^, whereas other substitutions (Fe_1_^(8c)^, Fe_1_^(32f)^ and Fe_1_^(48h)^) decrease the stability of Cr_22_Fe_1_C_6_. A similar result was reported by Fang *et al*.^37^. However, in contrast to the present work, previous work neglects important thermal excitations such as the effect of vibrational entropy on stability. This severely limits a comparison between previous ab initio results and experiment.

In this paper, we investigated the impact of site occupancy of Fe in the γ-Cr_23–*x*_Fe_*x*_C_6_ (*x = *0–3) carbide phase in detail using the cluster-expansion method (CEM) and the cluster-variation method (CVM) based on first-principles as function of composition and temperature. We found that if the partial site occupation is more properly taken into account in the cluster expansion, the preferred site occupancy is substantially different from the case when the cluster expansion mostly relies on full occupation configurations.

## Results and Discussion

### γ-Cr_23**−***x*_Fe_*x*_C_6_ (*x* = 0–3): super-ion cluster morphology

**Figure 2:**
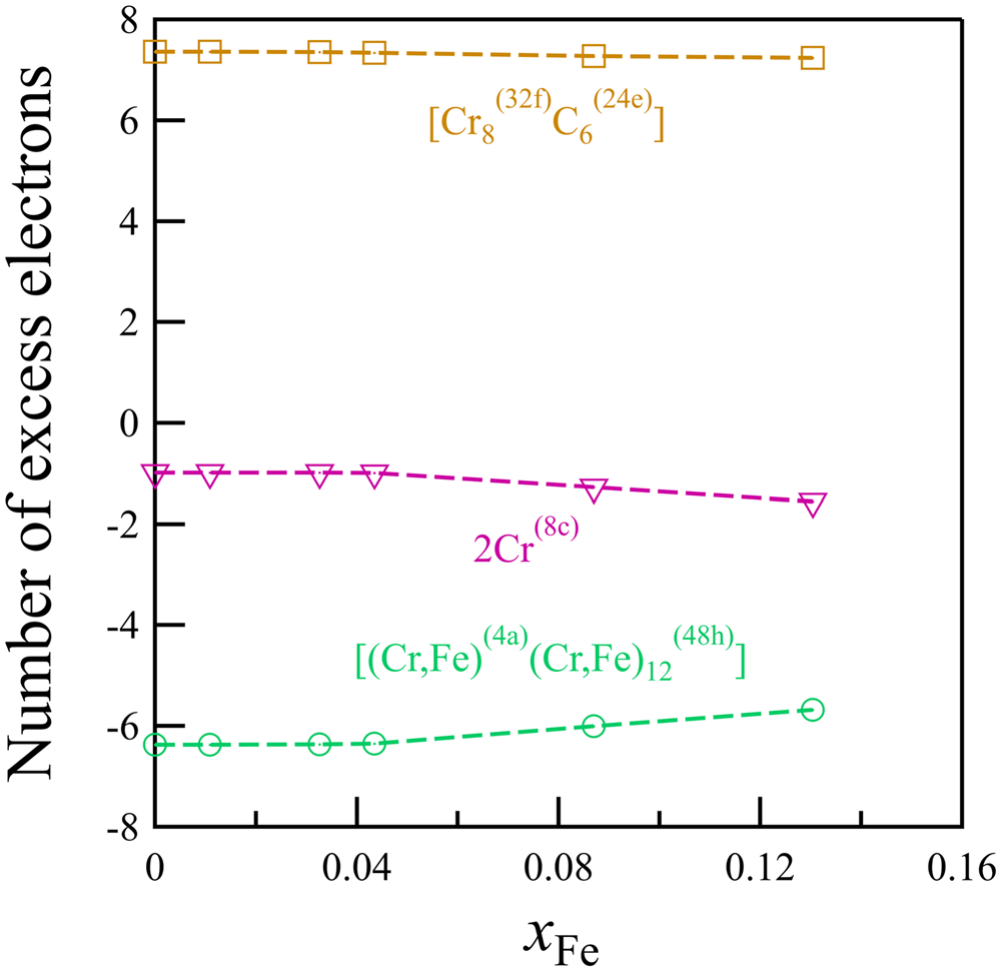
The number of excess electrons for the super-ions in γ-Cr_23−*x*_Fe_*x*_C_6_ as obtained from a Bader charge analysis^39,40^ as a function of *x*_Fe_ for the most stable configurations at *T* = 0 K.

**Figure 3:**
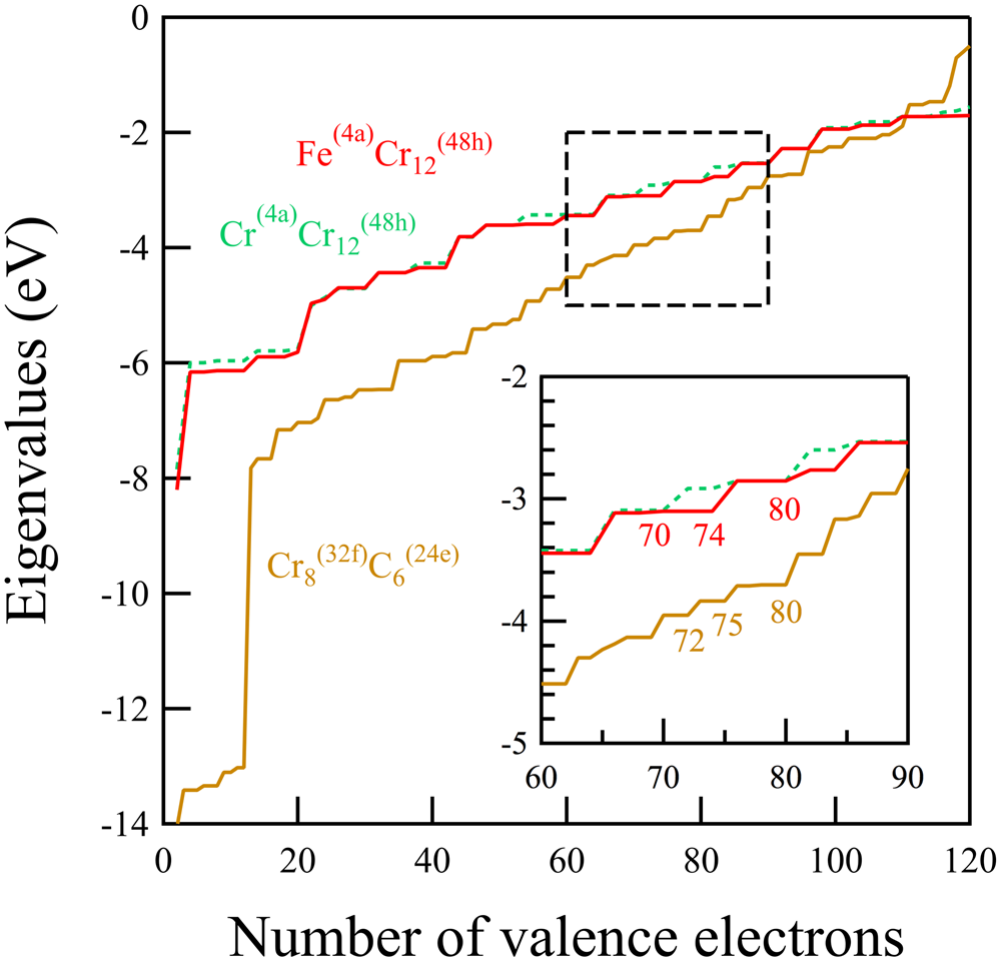
Computed eigenvalues for isolated neutral Cr_8_^(32f)^C_6_^(24e)^, Cr^(4a)^Cr_12_^(48h)^ and Fe^(4a)^Cr_12_^(48h)^ clusters.

Although subsequently forgotten, Westgren^27,38^ already recognized that the Cr_23_C_6_ structure can be viewed as composed of Cr^(4a)^Cr_12_^(48h)^ and Cr_8_^(32f)^C_6_^(24e)^ clusters in a NaCl arrangement with Cr^(8c)^ atoms as interstitials, see Fig. 1b. A Bader charge analysis^39,40^ coupled with an analysis of the electron levels of the isolated clusters supports the ionic NaCl interpretation of the structure, as evidenced by Figs 2 and 3. The Bader charges as a function of Fe fraction (*x*_Fe_ = *x*/23), shown in Fig. 2, reveal that Cr^(4a)^Cr_12_^(48h)^ is more than 6 electrons deficient, rather independent of Fe content, and Cr_8_^(32f)^C_6_^(24e)^ has more than 7 additional electrons, while the interstitial 8c sites balance the charges. As is typical of ionic configurations we expect to see a significant gap between the highest occupied and the lowest unoccupied molecular orbitals (HOMO–LUMO gap) for this charge state. Figure 3 confirms this: isolated Cr^(4a)^Cr_12_^(48h)^ features a large HOMO–LUMO gap for 70, 74, and 80 valence electrons, implying preferred charges of 8+, 4+, and 2−, the former corresponding nicely to the Bader charge of more than 6 electron deficient. The isolated Cr_8_^(32f)^C_6_^(24e)^ cluster has a large HOMO-LUMO gap for 72, 75, and 80 valence electrons, implying preferred charges of 0, 3−, and 8−, the latter agreeing well with the Bader charge of more than seven excess electrons.

The Fe-Cr substitution behavior can be understood in terms of the HOMO-LUMO gaps of the Cr^(4a)^Cr_12_^(48h)^ and Cr_8_^(32f)^C_6_^(24e)^ super-ions. When some Fe atoms substitute for Cr atoms on the 48h sites in the Cr^(4a)^Cr_12_^(48h)^ or on the 32f sites in the Cr_8_^(32f)^C_6_^(24e)^ super-ions, the high symmetry of the super-ions is lost. Such symmetry breaking lowers the degeneracies of electron levels and therefore also reduces the HOMO-LUMO gap. Large HOMO-LUMO gaps indicate favorable electronic configurations. Therefore, Fe substitutions on 48h or 32f sites destabilize the super-ions and thus the whole carbide structure. When Fe substitutes for Cr on the 4a site in Cr^(4a)^Cr_12_^(48h)^, or Fe substitutes Cr on the 8c sites, the symmetry remains exactly the same, the favorable large HOMO-LUMO gap of the super-ions is maintained, and the structure remains stable. Therefore, incorporation of Fe in Cr_23_C_6_ is energetically preferred on the 4a and 8c sites.

### Free energy and vibrational entropy contribution

Consideration of the vibrational effect in the free energy calculations can significantly alter the predicted carbide phase stability obtained at *T* = 0 K. Indeed, the vibrational free energy contributions can cause a re-ranking of the most stable structures (Table S1) in the lowest energy search. In this regard, the phase stability of the precipitates can be deduced by accounting for the enthalpy of formation, whereby the lattice vibration of the compounds is also included in the total free energy using CVM calculations within the Debye model^41,42^. The configurations studied for the full and partial occupations are summarized in Table S2 in the Supplementary information. Each structure is denoted in the form MMMM where sequentially reference is made to the 4a, 8c, 32f, 48h sites and M = −(Fe) when a site is occupied by Cr (partially by Fe). Subscript for Fe indicates fraction Fe atoms with remaining fraction Cr. We used the Debye model because a full phonon calculation was not feasible for all structures. Our method for obtaining the Debye temperature is described in detail in the supplementary information (see Table S2). The free energy of a supercell, $\tilde{G}(T)$, is obtained by adding the temperature dependent vibrational free energy, *G*_Debye_(*T*), to the ab initio computed ground state enthalpy, *H*,

1 $$\tilde{G}(T)=H+{G}_{{\rm{Debye}}}(T),$$

where *T* is the absolute temperature. The tilde superscript is a reminder that the configurational entropy term has not been accounted for. The Debye vibrational free energy^43–45^ is computed with

2 $${G}_{{\rm{Debye}}}=\frac{9}{8}{k}_{{\rm{B}}}{\theta }_{{\rm{D}}}+{k}_{{\rm{B}}}T\times [3\,\mathrm{ln}(1-{e}^{-{\theta }_{{\rm{D}}}/T})-{{\rm{D}}}_{3}(\frac{{\theta }_{{\rm{D}}}}{T})]$$

where *k*_B_ is Boltzmann’s constant, *θ*_D_ is the Debye temperature, and D_3_ is the Debye integral.

The first term on the right-hand side denotes the zero-point vibrational contribution and the second indicates the temperature dependent vibrational contribution.

The formation free energies of the perfectly periodic supercells, ${\rm{\Delta }}{\tilde{G}}_{{\rm{f}}}(T)$, without including the entropic contribution from configurational disorder, can then be defined with respect to the terminal carbides as

3 $${\rm{\Delta }}{\tilde{G}}_{{\rm{f}}}(T)[{{\rm{Cr}}}_{23-x}{{\rm{Fe}}}_{x}{{\rm{C}}}_{6}]=\tilde{G}(T)[{{\rm{Cr}}}_{23-x}{{\rm{Fe}}}_{x}{{\rm{C}}}_{6}]-\{\frac{23-x}{23}\tilde{G}(T)[{{\rm{Cr}}}_{23}{{\rm{C}}}_{6}]+\frac{x}{23}\tilde{G}(T)[{{\rm{Fe}}}_{23}{{\rm{C}}}_{6}]\}$$

where the composition of the supercell is written in square brackets.

The free energies, ${\rm{\Delta }}{\tilde{G}}_{{\rm{f}}}(T)$, are used in a cluster expansion to yield temperature dependent effective cluster interactions. The effective cluster interactions are used in the CVM to obtain the formation free energy, Δ*G*_f_(*T*), with full inclusion of configurational entropy and configurational enthalpy associated with temperature dependent partial site occupancy. The thermodynamically favored site occupation is determined by minimizing Δ*G*_f_(*T*). In our calculations, we have neglected the effects of thermal volume expansion.

**Figure 4:**
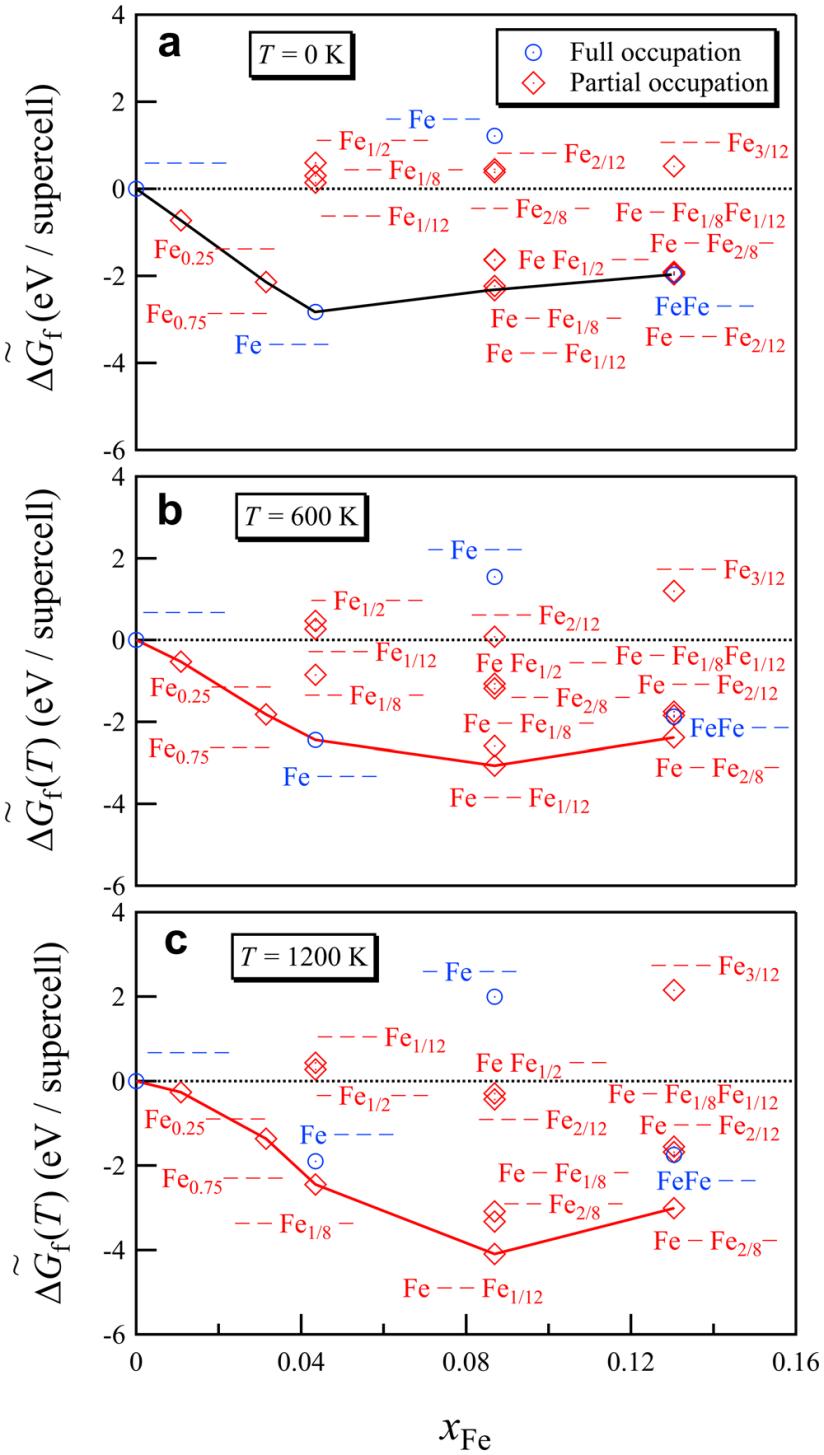
The free energy of formation, ${\rm{\Delta }}{\tilde{G}}_{{\rm{f}}}$, of γ-Cr_23−*x*_Fe_*x*_C_6_ (*x* = 0–3) as a function of *x*_Fe_ at (**a**) *T* = 0 K (**b**) *T* = 600 K and (**c**) *T* = 1200 K. The open circles (diamonds) indicate the results from full (partial) site occupation structures.

Figure 4 shows ${\rm{\Delta }}{\tilde{G}}_{{\rm{f}}}$ of the carbides as a function of *x*_Fe_ for the cases of both full and partial occupation at various temperatures. It is apparent that many Fe substituted carbides have lower energy than mixtures of the terminal carbides (Cr_23_C_6_ and Fe_23_C_6_) at the same composition. As a function of temperature, it is apparent that even when configurational entropy effects are ignored, the vibrational free energy contribution by itself causes shifts in the most favorable carbide structures: at *T = *0 K (Fig. 4a) the (Fe – – –) configuration is most favorable at *x*_Fe_ ≈ 0.043. At *T = *600 K (Fig. 4b), the same configuration remains favored, while at 1200 K (Fig. 4c), the partial occupancy (– – Fe_1/8_ –) configuration becomes preferred. At *x*_Fe_ ≈ 0.087, (Fe – Fe_1/8_ –) and (Fe – – Fe_1/12_) configurations have similar low free energies at *T* = 0 K. Vibrational free energy contributions increasingly favor the (Fe – – Fe_1/12_) configuration over the (Fe – Fe_1/8_ –) configuration as temperature is raised. When even more Fe atoms substitute for Cr (*x*_Fe_ ≈ 0.130), even more partial occupied configurations (Fe – Fe_1/8_ Fe_1/12_), (Fe – Fe_2/8_ –), and (Fe – – Fe_2/12_), have free energies very close to the fully occupied (Fe Fe – –) configuration at 0 K. However, at higher temperatures, the vibrational excitations cause a gradual preference for the (Fe – Fe_2/8_ –) configuration. This is not an isolated case, at higher temperatures several partial occupancy structures sink below the convex hull formed by full occupancy structures only. So a general trend emerges where vibrational excitations increasingly favor partial occupancy configurations over full occupancy configurations as temperature is raised. Clearly the vibrational effects by themselves already can produce shifts in site occupation behavior. The volume per atom decreases monotonically with *x*_Fe_, as shown in Fig. S1a. The atomic volumes of the most stable configurations were linearly fitted as (10.045 − 0.549 *x*_Fe_ [Å^3^]), where the negative slope is in agreement with the reported linear fits to experimental results of (10.439 − 0.574 *x*_Fe_ [Å^3^]) by Yakel^32^, (10.385 − 0.390 *x*_Fe_ [Å^3^]) by Shaw^32,46^, and (10.443 − 0.536 *x*_Fe_ [Å^3^]) by Westgren^32,47^. The computed atomic volumes are smaller than the reported experimental values. This is a well-known deficiency of the generalized gradient approximation (GGA) of the exchange-correlation functional^48^, especially for systems containing early 3d-elements^49,50^. For very Cr-rich carbides there is no net magnetization, however, already at an iron concentration *x*_Fe_ ≈ 0.043 a transition to the ferromagnetic state occurs, see Fig. S1b.

To understand the nature of chemical bonding in the most stable γ-Cr_23−*x*_Fe_*x*_C_6_ (*x* = 0–3) carbide structures, and the relevant changes with increased Fe content, we examined the electron localization function (ELF)^51^. ELF contour plots in the (110) plane of the carbide phase are displayed for four different Fe occupations, ranging from *x*_Fe_ = 0.0 to 0.130, see Fig. S2b. It is readily apparent that with increasing Fe content the character of bonding locally changes. While the ELF usually indicates degree of covalency, here it nicely illustrates changes in spin polarization. The arrows indicate the area in the bonding region between nearest neighbor 48h-48h Cr atoms perpendicular to the cross section plane where the spin polarization is strongly increased due to the presence of extra Fe atoms.

At *T* = 1200 K, ${\rm{\Delta }}{\tilde{G}}_{{\rm{f}}}$ of the most stable carbide at *x*_Fe_ ≈ 0.087 (Fe – – Fe_1/12_), was calculated as approximately −4.09 eV/supercell, quite comparable with the experimental enthalpy of −3.34 eV/supercell^52^ and higher than the value of −12.12 eV/supercell reported using a thermodynamic Calphad assessment of the Fe–Cr–C system^53^.

### Site occupation parameter

**Figure 5:**
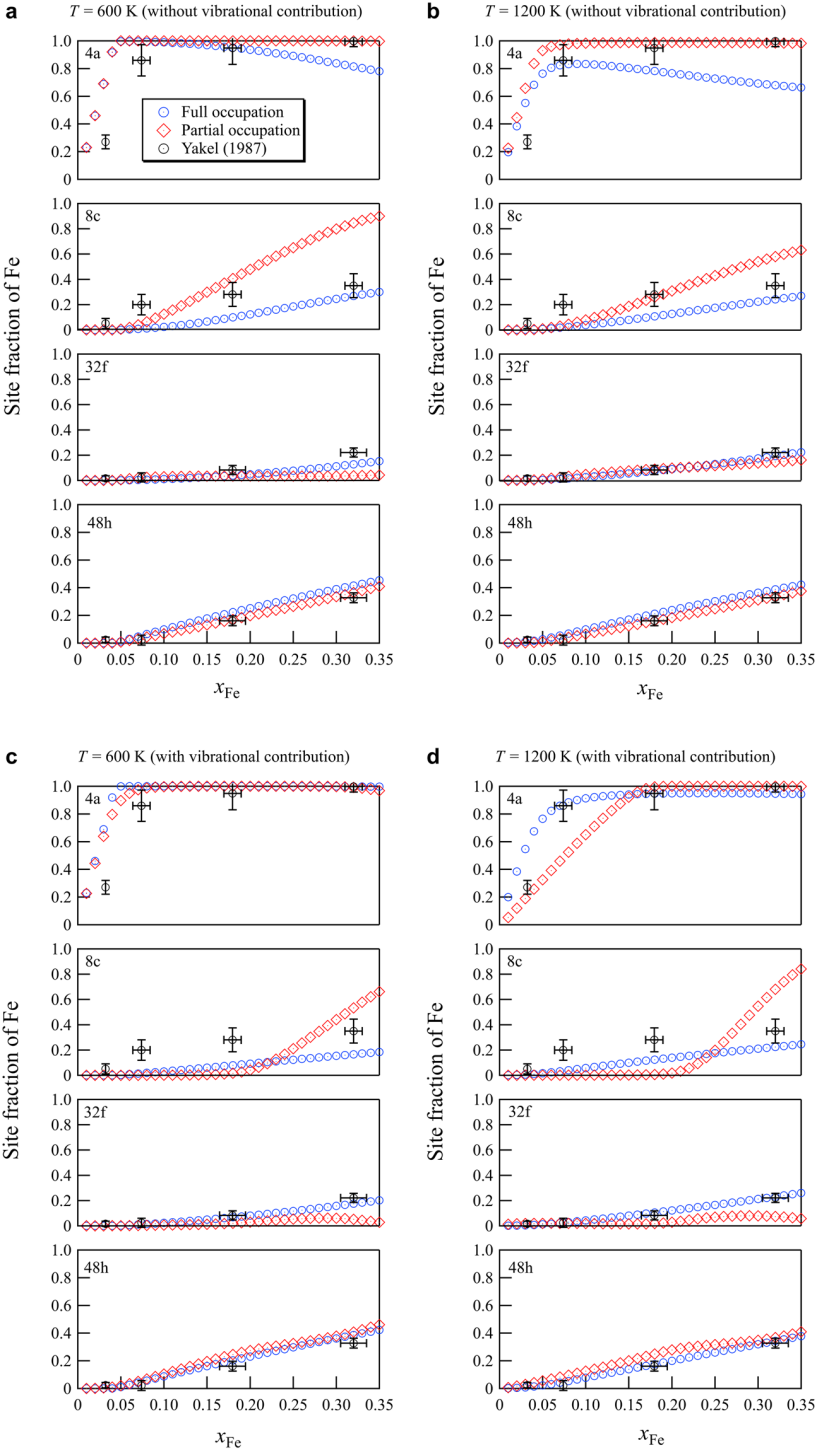
Iron site occupation on the inequivalent metal sites of γ-Cr_23−*x*_Fe_*x*_C_6_ (*x* = 0–3) as a function of *x*_Fe_, (**a**,**b**) without and (**c**,**d**) with vibrational effects at *T* = 600 K and *T* = 1200 K. The experimental data with error bars are plotted also for comparison^32^.

Finally, Fig. 5 shows the site fraction of Fe at each metal site computed with the CVM, both without (Fig. 5a,b) and with (Fig. 5c,d) inclusion of vibrational contributions. Experimental site occupations along with their error bars are also plotted for comparison^32^. In the results without the vibrational contribution, we found that inclusion of partial occupation structures in the cluster expansion gave improvement with experiment for the 4a and 48 h sites at both 600 K and 1200 K. However, for the 8c and 32f sites no such clear improvement could be seen. The results with vibrational contributions show more often that including the partial occupation structures in the cluster expansion does not improve the agreement with experimentally measured site occupations, the only exception to this being the 4a site at 600 K. The inclusion of vibrational effects seems to improve the agreement with experiment in particular for the results for the 4a site, for the other sites no such clear-cut conclusion can be drawn. Overall, we see a fair agreement with the experimental results, in particular for the 4a, 32f and 48h sites theoretical results are found within the experimental error bars. For the 8c sites agreement, in all theoretical treatments we considered is less good.

Fe is found to preferentially substitute for Cr at the 4a site, which is consistent with earlier work^34–37^. With increasing iron content, in contrast to the experimental findings, we find that the 4a sites saturate before the 8c sites take Fe occupancy. The Fe uptake in the 32f and 48h sites increases gradually with iron content, and in the 48h sites a little more rapidly than in the 32f sites, in good agreement with experiment^31,32^.

## Conclusion

In this work, we revived an old interpretation of the Cr_23_C_6_ structure as an NaCl arrangement of super-ions. We show that such super-ions can be recognized with approximate constant charges irrespective of composition, at least within the range 0 ≤ *x* ≤ 3 in Cr_23–*x*_Fe_*x*_C_6_. The super-ion description not only gives a rationalization for the crystal structure, but also gives an understanding for the site preference of Fe in Cr_23_C_6_. As this reasoning does not rely on the specifics of Fe, but merely assumes that elastic strain effects play an subordinate role, we expect that other alloying elements, with an atomic size comparable to Cr, can be predicted and understood as well.

It is also shown that including vibrational effects changes which structures populate the convex hull. With increasing temperature, it is seen that the vibrational free energy causes structures with partial site occupancy to sink below the convex hull formed by the full occupancy structures, even when configurational entropy effects are neglected. Of course, configurational free energy terms would further favor partial occupancy structures over full occupancy structures. This implies that vibrational effects cannot be ignored in the prediction of site preference.

Including partial occupancy structures in the cluster expansion gives significant changes in the site occupation. However, these changes did not give a univocal improvement with the experimental data. Inclusion of vibrational effects however, did give a systematic improvement of computed site occupancies with experimental results.

## Methods

### Cluster Expansion Method (CEM) & Cluster Variation Method (CVM)

**Figure 6:**
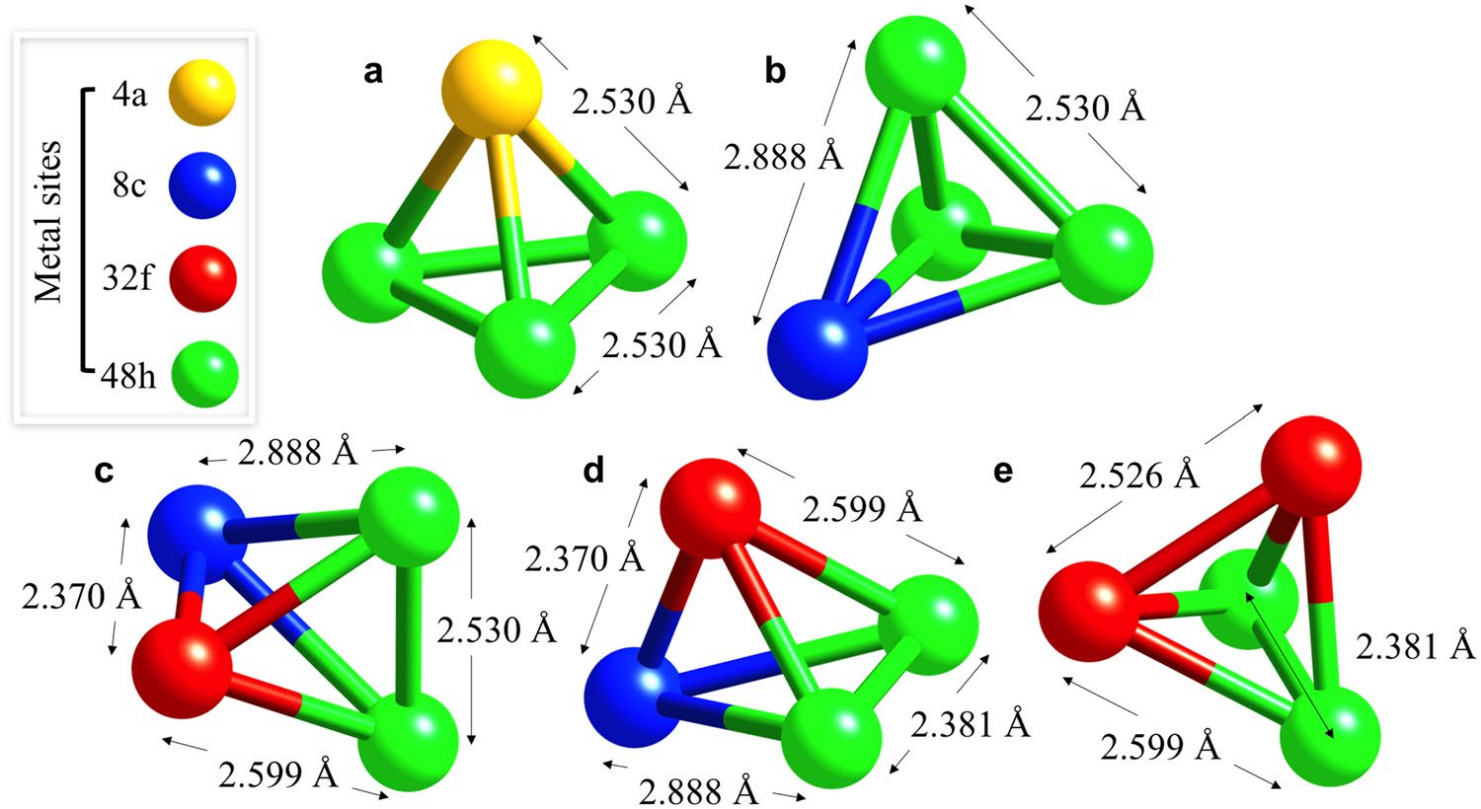
Five 4-site clusters selected for the CEM, namely, (**a**) M^(4a)^M_3_^(48h)^, (**b**) M^(8c)^M_3_^(48h)^, (**c**), (**d**) both M^(8c)^M^(32f)^M_2_^(48h)^ distinguished by their 48h-48h interatomic distance, and (**e**) M_2_^(32f)^M_2_^(48h)^. The interatomic distances are those obtained for Cr_23_C_6_ with *a*_0_ = 10.524 Å.

In this work, we have used CEM and CVM^54^ to study the effect of the partial occupation of inequivalent metallic sites in the γ-Cr_23−*x*_Fe_*x*_C_6_ (*x* = 0–3) carbide phase, where the interactions were used to compute the entropy and Gibbs energy of the phase as function of the temperature and composition within the tetrahedron approximation^21^. The cluster expansion was carried out using a pool of clusters generated by the condition that no two sites in a cluster are separated more than 0.31 times the M_23_C_6_ lattice parameter. As the lattice parameter is about 1 nm, this means no sites are further than about 0.3 nm apart. Using this criterion, five distinct types of 4-site clusters are the largest clusters that can be generated^55^. These clusters are shown in Fig. 6. Further details of the method are given in the Supplementary information.

### Ab initio calculations

As inputs for the CEM, we performed total energy calculations using pseudopotentials generated by the projected augmented wave (PAW) method as implemented in the Vienna Ab initio Simulation Package (VASP)^56,57^. The number of valence electrons are 4, 6, and 8 for C:(2s^2^2p^2^), Cr:(3d^5^4s^1^), and Fe:(3d^7^4s^1^), respectively. We considered collinear spin-polarization for all structures within the generalized gradient approximation (GGA) for the exchange–correlation functional as formulated by Perdew, Burke, and Ernzerhof (PBE)^48^. All of the calculations were performed using the supercell technique, where periodic boundary conditions and full relaxation of internal coordinates and lattice parameters were considered. The adopted supercell contains one conventional cubic unit cell consisting of 92 metal atoms and 24 carbon atoms. The cutoff energy for plane wave expansion was 520 eV and the k-point sampling grid was 7 × 7 × 7. The calculations were considered converged when the change of the total energy between successive self-consistency iterations reached 10^−5^ eV or less and simultaneously the magnitude of the greatest force on any atom was less than 10^−4^ eV/Å.

## Electronic supplementary material

Supplementary information
