# Supplementary material for: Effect of mixed partial occupation of metal sites on the phase stability of γ-Cr23−xFexC6 (x = 0–3) carbides
Source: Sci Rep. 2018 May 8;8:7279. doi: 10.1038/s41598-018-25642-y (PMC5940797; doi:10.1038/s41598-018-25642-y)
Supplement: Supplementary file 1 — Supplementary information [file 41598_2018_25642_MOESM1_ESM.pdf]

Effect of mixed partial occupation of metal sites on the phase stability of γ-$Cr_{23-x}Fe_xC_6$ (x = 0–3) carbides

Maaouia Souissi[1*], Marcel H. F. Sluiter[2], Tetsuya Matsunaga[1, 3], Masaaki Tabuchi[1], Michael J. Mills[3] & Ryoji Sahara[1]

[1] *Research Center for Structural Materials, National Institute for Materials Science, 1-2-1 Sengen, Tsukuba, Ibaraki 305-0047, Japan*

[2] *Department of Materials Science and Engineering, Delft University of Technology, Mekelweg 2, 2628 CD Delft, The Netherlands*

[3] *Department of Materials Science and Engineering, The Ohio State University, 478 Watts Hall, 2041 College Rd, Columbus, OH 43210, USA*

*Correspondence should be addressed to M. Souissi

(email: SOUISSI.Maaouia@nims.go.jp)

Table S1. Description of the energetically most stable structures γ-$Cr_{23-x}Fe_xC_6$ (x = 0–3). Wyckoff sites, atomic occupancy, and fractional coordinates are specified. Each structure is denoted in the form MMMM where sequentially reference is made to the 4a, 8c, 32f, 48h sites and M = −(Fe) when a site is occupied by Cr (partially by Fe). Subscript for Fe indicates fraction Fe atoms with the remaining fraction Cr atoms.

Structure		Formula	Wyckoff site	Species	Sites (fractional coordinates)
(− − − −)	Theory	$Cr_{23}C_6$	4a	Cr	(0,0,0)
			8c	Cr	(0.250,0.250,0.250)
			32f	Cr	(0.380,0.380,0.380)
			48h	Cr	(0,0.169,0.169)
			24e	C	(0.276,0,0)
	Expt.		4a[1]	Cr	(0,0,0)
			8c[1]	Cr	(0.250,0.250,0.250)
			32f[1]	Cr	(0.385,0.385,0.385)
			48h[1]	Cr	(0,0.165,0.165)
			24e[1]	C	(0.275,0,0)
($Fe_{0.25}$ − − −)		$Cr_{22.75}Fe_{0.25}C_6$	4a	Fe	(0,0,0)
				Cr	(0,0.5,0.5), (0.5,0,0.5), (0.5,0.5,0)
			8c	Cr	(0.250,0.250,0.250)
			32f	Cr	(0.381,0.381,0.381)
			48h	Cr	(0,0.168,0.168)
			24e	C	(0.277,0,0)
($Fe_{0.75}$ − − −)		$Cr_{22.25}Fe_{0.75}C_6$	4a	Fe	(0,0.5,0.5), (0.5,0,0.5), (0.5,0.5,0)
				Cr	(0,0,0)
			8c	Cr	(0.249,0.249,0.249)
			32f	Cr	(0.380,0.380,0.380)
			48h	Cr	(0,0.169,0.169)
			24e	C	(0.276,0,0)
(Fe − − −)		$Cr_{22}Fe_1C_6$	4a	Fe	(0,0,0)
			8c	Cr	(0.250,0.250,0.250)

		32f	Cr	(0.380,0.380,0.380)
		48h	Cr	(0,0.167,0.167)
		24e	C	(0.277,0,0)
(Fe − − $Fe_{1/12}$)	$Cr_{21}Fe_2C_6$	4a	Fe	(0,0,0)
		8c	Cr	(0.249,0.249,0.249)
		32f	Cr	(0.380,0.379,0.380)
		48h	Fe	(0.5,0.331,0.166) (0.166,0.331,0.5)
				(0.5,0.331,0.833) (0.833,0.331,0.5)
			Cr	Remaining 44 sites
		24e	C	(0.278,0,0)
(Fe − − $Fe_{2/12}$)	$Cr_{20}Fe_3C_6$	4a	Fe	(0,0,0)
		8c	Cr	(0.249,0.249,0.250)
		32f	Cr	(0.380,0.379,0.380)
		48h	Fe	(0.332,0.667,0) (0.669,0.334,0)
				(0.667,0.667,0) (0.330,0.334,0)
				(0.5,0.332,0.168) (0.166,0.331,0.5)
				(0.5,0.332,0.831) (0.833,0.331,0.5)
			Cr	Remaining 40 sites
		24e	C	(0.278,0,0)

[1]Bowman, A. L., Arnold, G. P., Storms E. K. & Nereson, N. G. The crystal structure of $Cr_{23}C_6$. *Acta Cryst.* B**28**, 3102–3103 (1972).

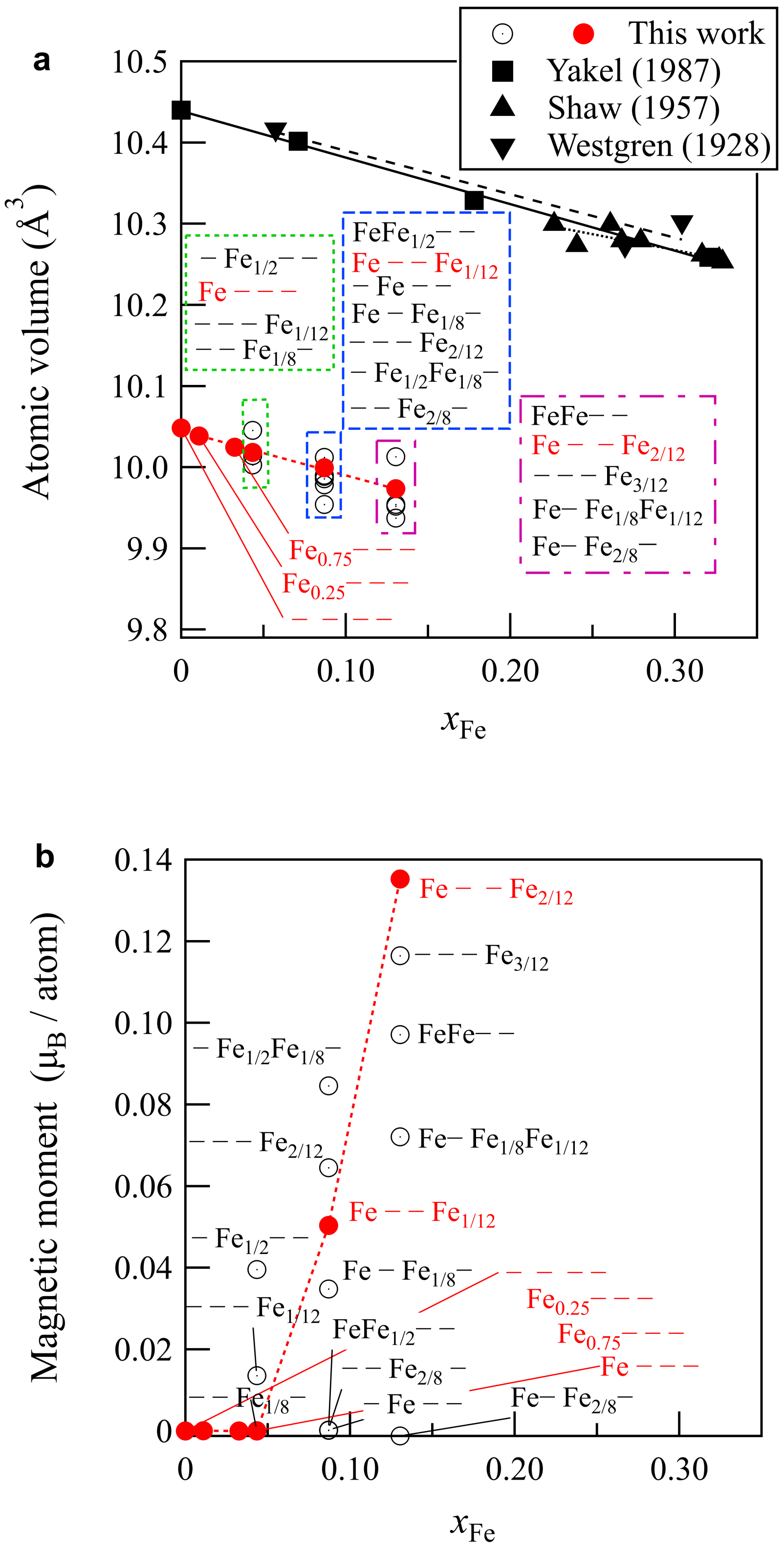

Figure S1. (a) Per atom volume (Å^3) and (b) per atom magnetic moment (μ_B / atom) of γ-$Cr_{23-x}Fe_xC_6$ (x = 0–3) as a function of x_{Fe}= x/23 and partial site occupation. Adopted notation for each structure is as indicated in Table S1. In panel (a) the configurations with the same Fe content are sorted in boxes in downward descending order.

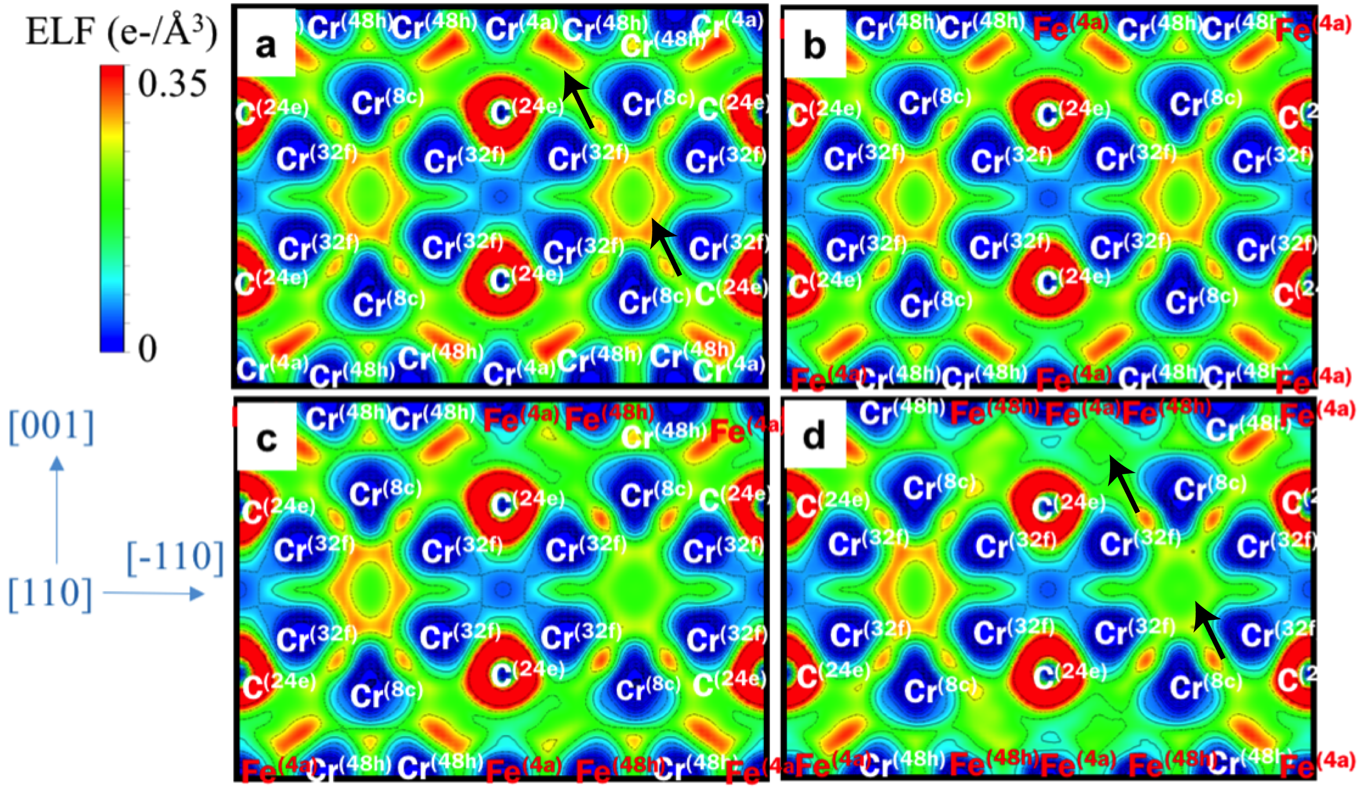

Figure S2. Contour plots of the electron localization function (ELF) in the (110) plane for four different Fe fractions, namely, (a) $Cr_{23}C_6$ (x_{Fe} = 0), (b) Fe – – – (x_{Fe} = 0.043), (c) Fe – – $Fe_{1/12}$ (x_{Fe} = 0.087), and (d) Fe – – $Fe_{2/12}$ (x_{Fe} = 0.130). The red(blue) zones indicate maximum(minimum) spin compensation. The values of ELF range between 0 and 0.35. The arrows indicate the area in the bonding region between nearest neighbor 48h-48h Cr atoms perpendicular to the cross section plane where the spin polarization is strongly increased due to the presence of extra Fe atoms.

1. Details of the cluster expansion method

For our cluster expansion, we first defined an initial pool of clusters. This cluster pool consisted of all clusters in which no two sites are farther apart than 0.31 a_0, where a_0 is the lattice parameter of the $M_{23}C_6$ carbide. This yields as largest possible clusters five 4-body clusters. Of course, all the subclusters are considered as well, such as four single-point clusters, seven pair clusters, and eight 3-body clusters, yielding a total of 24 clusters. When the actual cluster expansion was carried out it was found that the predictive error, or leave-one-out cross validation score, was lowest when two of the 4-body terms were excluded, thus yielding a cluster expansion with 22 rather than 24 effective cluster interactions. The 24e carbon occupied sites were not included in the cluster expansion because there is no disorder on the 24e sites. The formation free energies (meV/atom) of carbide phases obtained from DFT, and CEM together with their corresponding error, $\Delta\widetilde{G}_{\mathrm{f}}(T=0\ \mathrm{K})[\mathrm{CEM}]-\Delta\widetilde{G}_{\mathrm{f}}(T=0\ \mathrm{K})[\mathrm{DFT}]$, are shown in Fig. S3. CEM and DFT energy values differ by less than 15 meV/atom. In the cluster variation method calculations, the entropy formula was generated using all five 4-body clusters. We paid special attention to higher symmetry distributions of Fe atoms because often such highly symmetric configurations are extremes of energy, e.g., B2 (CsCl type) ordering on the bcc lattice often represents either the most negative[1,2], or the most positive enthalpy of formation in a bcc alloy[3]. By considering a few of the most symmetric configurations we can explore the energy range over which at a given composition energies are likely to vary. An additional benefit of this approach is that it limits the overwhelmingly large number of possible configurations with partial occupancy. At first, we selected a few Fe arrangements on each of the inequivalent sites with high symmetry constructed by up to four Fe atoms. The combination of the arrangements gives the Fe fraction, x_{Fe}, of 0.043 (x = 1), 0.087 (x = 2) and 0.130 (x = 3).

[1]Drautz, R., Díaz-Ortiz, A., Fähnle, M. & Dosch, H. Ordering and Magnetism in Fe-Co: Sequence of Ground-State Structures. *Phys. Rev. Lett.* **93**, 067202 (2004).
[2]Blum, V. & Zunger, A. Structural complexity in binary bcc ground states: The case of bcc Mo-Ta. *Phys. Rev. B*. **69**, 020103(R) (2004).
[3]Feng R., Liaw P.K., Gao M.C. & Widom M. First-principles prediction of high-entropy-alloy stability, *npj Comput. Mater.* **3**, 50 (2017).

2. Thermodynamic parameters derived using phonon calculations and Debye model.

The configurations studied, together with the thermodynamic parameters are listed in Table S2. For structures with low symmetry shown in blue italic type, the Debye temperature was estimated from a quadratic function considering the bulk modulus, cell volume, composition, and their cross terms.

The quadratic function was obtained by fitting to the Debye temperatures of 23 structures for which a full ab initio phonon calculation was feasible, where the harmonic heat capacity was used to extract the Debye temperature.

Table S2. Properties needed for the Debye model for configurations with both full and partial occupations: Debye temperature θ_D (K), equilibrium lattice parameter a_0 (Å), per atom volume V_0 (Å^3), pressure derivative of the volume dV/dP, bulk modulus B_0 (GPa), and pressure derivative of the bulk modulus at zero pressure B'_0. Notation for each configuration is as indicated in Table S1. For structures shown in blue italic type, the Debye temperature was estimated from a 10-parameter quadratic function of B_0, V_0, composition, and their cross terms. Structures with $B'_0 = 4.0$ are estimated with a second-order Birch–Murnaghan equation of state. Such a second-order fit always yields $B'_0 = 4.0$.

Structure	Formula	Number of Cr	θ_D (K)	a_0 (Å)	V_0 (Å^3)	dV/dP	B_0 (GPa)	B'_0
Full occupation								
(Fe Fe Fe Fe)	*$Fe_{23}C_6$*	*0*	*448.2*	*10.308*	*9.615*	*−0.035*	*280.7*	*4.000*
(– Fe Fe Fe)	*$Cr_1Fe_{22}C_6$*	*1*	*482.4*	*10.473*	*9.717*	*−0.034*	*275.5*	*4.000*
(Fe – Fe Fe)	*$Cr_2Fe_{21}C_6$*	*2*	*493.8*	*10.524*	*9.706*	*−0.038*	*253.7*	*4.000*
(– – Fe Fe)	$Cr_3Fe_{20}C_6$	3	487.2	10.406	9.701	−0.047	220.0	3.721
(Fe Fe – Fe)	$Cr_8Fe_{15}C_6$	8	538.8	10.459	9.814	−0.044	370.8	4.000
(– Fe – Fe)	$Cr_9Fe_{14}C_6$	9	514.4	10.460	9.888	−0.041	227.8	5.615
(Fe – – Fe)	$Cr_{10}Fe_{13}C_6$	10	529.2	10.448	9.831	−0.041	241.5	4.000
(– – – Fe)	$Cr_{11}Fe_{12}C_6$	11	529.4	10.456	9.871	−0.038	244.3	4.823
(Fe Fe Fe –)	$Cr_{12}Fe_{11}C_6$	12	518.4	10.427	9.824	−0.047	221.1	4.000
(– Fe Fe –)	$Cr_{13}Fe_{10}C_6$	13	531.8	10.425	9.811	−0.040	201.5	4.000
(Fe – Fe –)	$Cr_{14}Fe_9C_6$	14	512.4	10.392	9.702	−0.031	280.3	4.000
(– – Fe –)	$Cr_{15}Fe_8C_6$	15	522.0	10.401	9.722	−0.031	293.5	4.000
(Fe Fe – –)	$Cr_{20}Fe_3C_6$	20	546.0	10.487	10.011	−0.032	393.6	4.000
(– Fe – –)	*$Cr_{21}Fe_2C_6$*	*21*	*561.7*	*10.521*	*10.048*	*−0.035*	*238.6*	*4.000*
(Fe – – –)	$Cr_{22}Fe_1C_6$	22	570.0	10.513	10.030	−0.032	299.1	4.406
(– – – –)	$Cr_{23}C_6$	23	558.0	10.524	10.077	−0.033	300.6	4.468
Partial occupation								
(– – – $Fe_{3/12}$)	*$Cr_{20}Fe_3C_6$*	*20*	*572.3*	*10.510*	*10.022*	*−0.033*	*284.9*	*4.818*
(Fe – $Fe_{2/8}$ –)	$Cr_{20}Fe_3C_6$	20	523.2	10.484	9.963	−0.033	295.8	3.917
(Fe – $Fe_{1/8}Fe_{1/12}$)	$Cr_{20}Fe_3C_6$	20	548.4	10.487	9.977	−0.034	274.5	4.000
(Fe – – $Fe_{2/12}$)	*$Cr_{20}Fe_3C_6$*	*20*	*547.1*	*10.497*	*9.999*	*−0.034*	*277.5*	*4.000*

(– – $Fe_{2/8}$ –)	$Cr_{21}Fe_2C_6$	21	484.8	10.495	9.991	−0.032	300.8	4.218
(– – – $Fe_{2/12}$)	*$Cr_{21}Fe_2C_6$*	*21*	*531.6*	*10.502*	*10.013*	*−0.034*	*308.1*	*4.578*
(Fe – $Fe_{1/8}$ –)	$Cr_{21}Fe_2C_6$	21	532.0	10.501	10.011	−0.034	287.9	4.000
(Fe – – $Fe_{1/12}$)	$Cr_{21}Fe_2C_6$	21	516.4	10.503	10.015	−0.033	301.2	4.000
(Fe $Fe_{1/2}$ – –)	$Cr_{21}Fe_2C_6$	21	572.0	10.514	10.051	−0.034	291.0	5.639
($Fe_{0.25}$ – – –)	$Cr_{22.25}Fe_{0.75}C_6$	22.25	568.4	10.515	10.052	−0.033	303.1	4.686
($Fe_{0.75}$ – – –)	$Cr_{22.75}Fe_{0.25}C_6$	22.75	565.2	10.520	10.068	−0.033	332.5	5.030
(– – $Fe_{1/8}$ –)	$Cr_{22}Fe_1C_6$	22	504.8	10.507	10.034	−0.033	334.7	5.883
(– – – $Fe_{1/12}$)	$Cr_{22}Fe_1C_6$	22	558.0	10.511	10.036	−0.033	299.5	4.809
(– $Fe_{1/2}$ – –)	$Cr_{22}Fe_1C_6$	22	546.8	10.513	10.046	−0.033	301.5	4.669

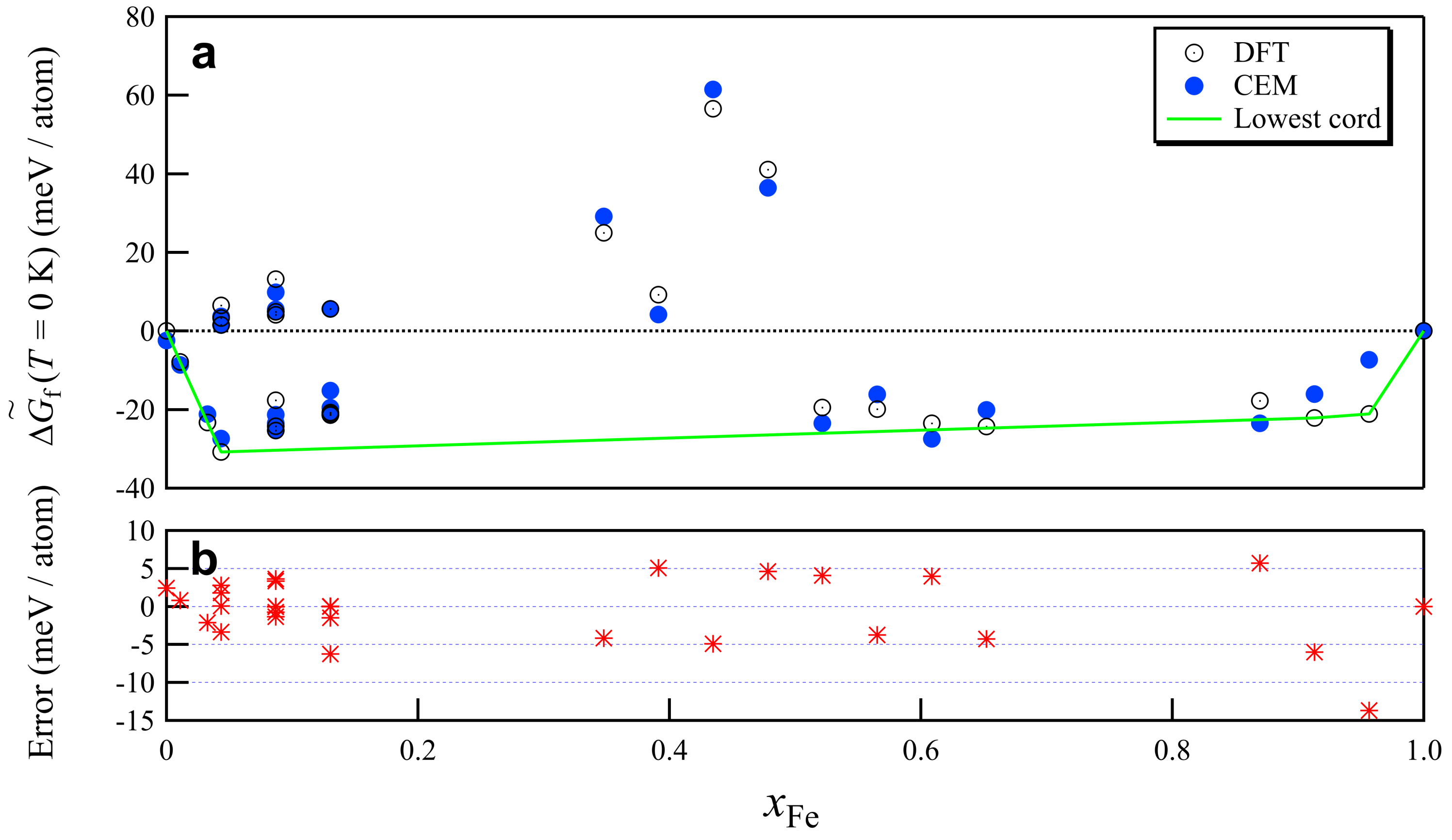

Figure S3. (a) The formation free energies, $\Delta\widetilde{G}_f(T = 0\ \mathrm{K})$ (meV/atom), of carbide phases obtained from DFT, and CEM. The convex hull with regard to the DFT values is indicated with the solid green line. The difference between DFT and CEM energy values, $\mathrm{error} = \Delta\widetilde{G}_f(T = 0\ \mathrm{K})[\mathrm{CEM}] - \Delta\widetilde{G}_f(T = 0\ \mathrm{K})[\mathrm{DFT}]$ (meV/atom), is shown in panel (b).
